# Diffuse midline glioma, H3 K27-altered, forming a suprasellar mass: A case report

**DOI:** 10.1016/j.radcr.2026.08.041

**Published:** 2026-09-11

**Authors:** Rihito Nakazato, Tetsuya Shimizu, Akihiko Teshigawara, Miku Fukasawa, Akira Baba, Satoshi Matsushima, Taku Gomi, Mayuko Terayama, Ai Iwauchi, Nei Fukasawa, Toshihide Tanaka, Hiroya Ojiri

**Affiliations:** a Department of Radiology, The Jikei University School of Medicine, Tokyo, Japan; b Department of Neurosurgery, The Jikei University School of Medicine, Kashiwa Hospital, Chiba, Japan; c Department of Pathology, The Jikei University School of Medicine, Tokyo, Japan; d Department of Radiology, The Jikei University Kashiwa Hospital, Chiba, Japan; e Department of Neurosurgery, The Jikei University School of Medicine, Tokyo, Japan

**Keywords:** Diffuse midline glioma H3 K27-altered, Suprasellar tumor, Optic chiasm, Visual deterioration, Hypothalamus

## Abstract

Diffuse midline glioma, H3 K27-altered (DMG), is an extremely aggressive tumor that mainly arises in midline structures such as the thalamus, brainstem, and spinal cord in children and young adults. We report a rare adult case of DMG presenting as a suprasellar mass. A 56-year-old man presented with progressive visual impairment over 2 months. Magnetic resonance imaging (MRI) revealed a lobulated mass centered on the optic chiasm and extending from the suprasellar region to the anterior aspect of the midbrain, with involvement of both optic tracts and the hypothalamus. Subtotal resection was performed, and histopathological and molecular genetic examination demonstrated a diffuse glioma harboring an H3F3A p.K28M (H3.3 K27M) mutation, leading to the diagnosis of diffuse midline glioma, H3 K27-altered (H3.3 K27M-mutant subtype), central nervous system WHO grade 4. Based on the clinical presentation and MRI findings, the tumor was considered to originate from the optic chiasm. However, autopsy findings suggested that the tumor likely originated from the hypothalamus and secondarily involved the optic chiasm. Despite postoperative chemoradiotherapy, the patient died approximately 5 months after symptom onset. Although DMG occurring in the suprasellar region with optic tract involvement is very rare, it should be considered in the differential diagnosis of suprasellar masses.

## Introduction

Diffuse midline glioma, H3 K27-altered (DMG), is a highly aggressive malignancy classified as a pediatric-type diffuse high-grade glioma, central nervous system (CNS) WHO grade 4 [1]. This entity is defined by the loss of histone H3 lysine 27 trimethylation (H3K27me3), primarily driven by the H3 K27M mutation, which leads to profound epigenetic dysregulation and tumorigenesis [1]. DMGs typically occur in midline structures such as the brainstem, thalamus, and spinal cord in children and young adults [2]. Herein, we report a rare adult case of DMG presenting as a suprasellar mass primarily involving the optic chiasm and hypothalamus.

## Case report

A 56-year-old man was noted to have decreased visual acuity (0.6 in the right eye [OD] and 0.7 in the left eye [OS]) during a routine medical checkup 6 months before presentation to our institution, although he reported no subjective visual symptoms at that time. Four months later, 2 months prior to presentation, he noticed progressive visual impairment. Two weeks before presenting to our institution, he visited the ophthalmology department at another hospital, where an intracranial lesion was suspected, and was subsequently referred to our institution. Ophthalmologic examination revealed right homonymous hemianopsia, and he was admitted for further evaluation and treatment. At presentation, visual acuity was 0.1 OD and 0.4 OS. Color vision was preserved, and no relative afferent pupillary defect was observed. Apart from the visual disturbance, no other cranial nerve deficits or hypothalamic dysfunction were noted. His medical history included hypertension, dyslipidemia, fatty liver disease, hyperuricemia, and obstructive sleep apnea. His family history was unremarkable. Routine hematologic and biochemical test results were within normal limits.

**Fig. 1:**
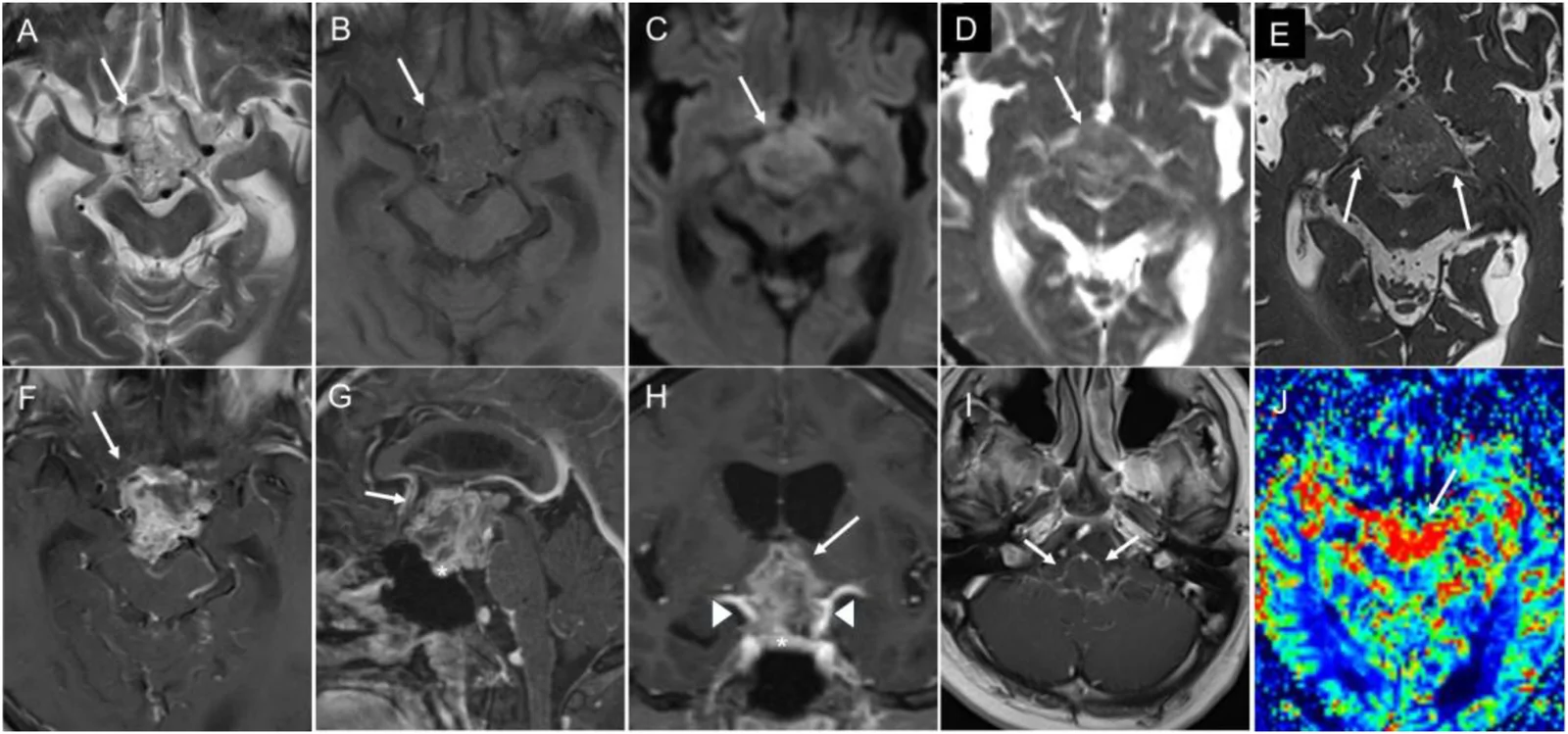
Brain MRI demonstrates a lobulated mass centered in the suprasellar region. The lesion shows heterogeneous hyperintensity with cystic components on T2-weighted images (A, arrow) and heterogeneous hypointensity on T1-weighted images (B, arrow). Diffusion-weighted imaging (C, arrow) shows peripheral hyperintensity with partially decreased ADC values (D); the ADC value is 0.98 × 10^−3^ mm^2^/s (D, arrow). A 3D T2-weighted image (SPACE) demonstrates extension along the optic tracts from the optic chiasm (E, arrows). Contrast-enhanced T1-weighted images (F, axial; G, sagittal; H, coronal) show heterogeneous enhancement with central nonenhancing areas (F-H, arrows). On the coronal image (H), the optic chiasm (arrowhead) is involved by the mass. No abnormalities are observed in the pituitary gland (G and H, *). Leptomeningeal enhancement is observed along the surface of the brainstem (I, arrow). Perfusion imaging shows elevated rCBV (J, arrow). Abbreviations: ADC, apparent diffusion coefficient; rCBV, relative cerebral blood volume; SPACE, sampling perfection with application optimized contrasts using different flip angle evolutions.

Approximately 3 weeks after presentation, brain magnetic resonance imaging (MRI) demonstrated a lobulated mass predominantly located in the suprasellar region, extending into the prepontine cistern and just reaching the anterior surface of the brainstem. The lesion was centered on the optic chiasm, extended posteriorly along both optic tracts, and involved the hypothalamus superiorly. The lesion was heterogeneously hyperintense on T2-weighted and FLAIR images and showed focal cystic changes. Diffusion-weighted imaging showed peripheral hyperintensity, and the apparent diffusion coefficient (ADC) was partially decreased, measuring 0.98 × 10^−3^ mm^2^/s. Contrast-enhanced T1-weighted images demonstrated heterogeneous enhancement with central nonenhancing areas. Perfusion imaging showed elevated relative cerebral blood volume (rCBV), consistent with increased intratumoral perfusion. The pituitary gland appeared normal. Leptomeningeal enhancement along the surface of the brainstem raised concern for meningeal dissemination (Fig. 1).

Because of the rapid progression of his symptoms, an urgent craniotomy for subtotal tumor resection was performed the following day. Preoperative imaging suggested that the tumor was highly vascular and encased the internal carotid artery; therefore, priority was given to obtaining tissue for histopathologic diagnosis rather than radical resection. Intraoperatively, the tumor appeared as a grayish, highly hemorrhagic lesion and was identified both lateral and medial to the right optic nerve. An adequate amount of tissue was obtained for diagnosis, and the operation was concluded.

Based on the diagnosis of a high-grade glioma, postoperative concurrent chemoradiotherapy with temozolomide (75 mg/m^2^/d) and cranial intensity-modulated radiation therapy (IMRT) was initiated approximately 3 weeks after surgery. The planned radiation dose was 60 Gy in 30 fractions; however, treatment was discontinued at 52 Gy in 26 fractions because of rapid disease progression. The patient died approximately 5 months after symptom onset.

Shortly before death, the patient developed a high fever of unclear etiology. No definitive source of the fever was identified on CT.

## Histological findings

**Fig. 2:**
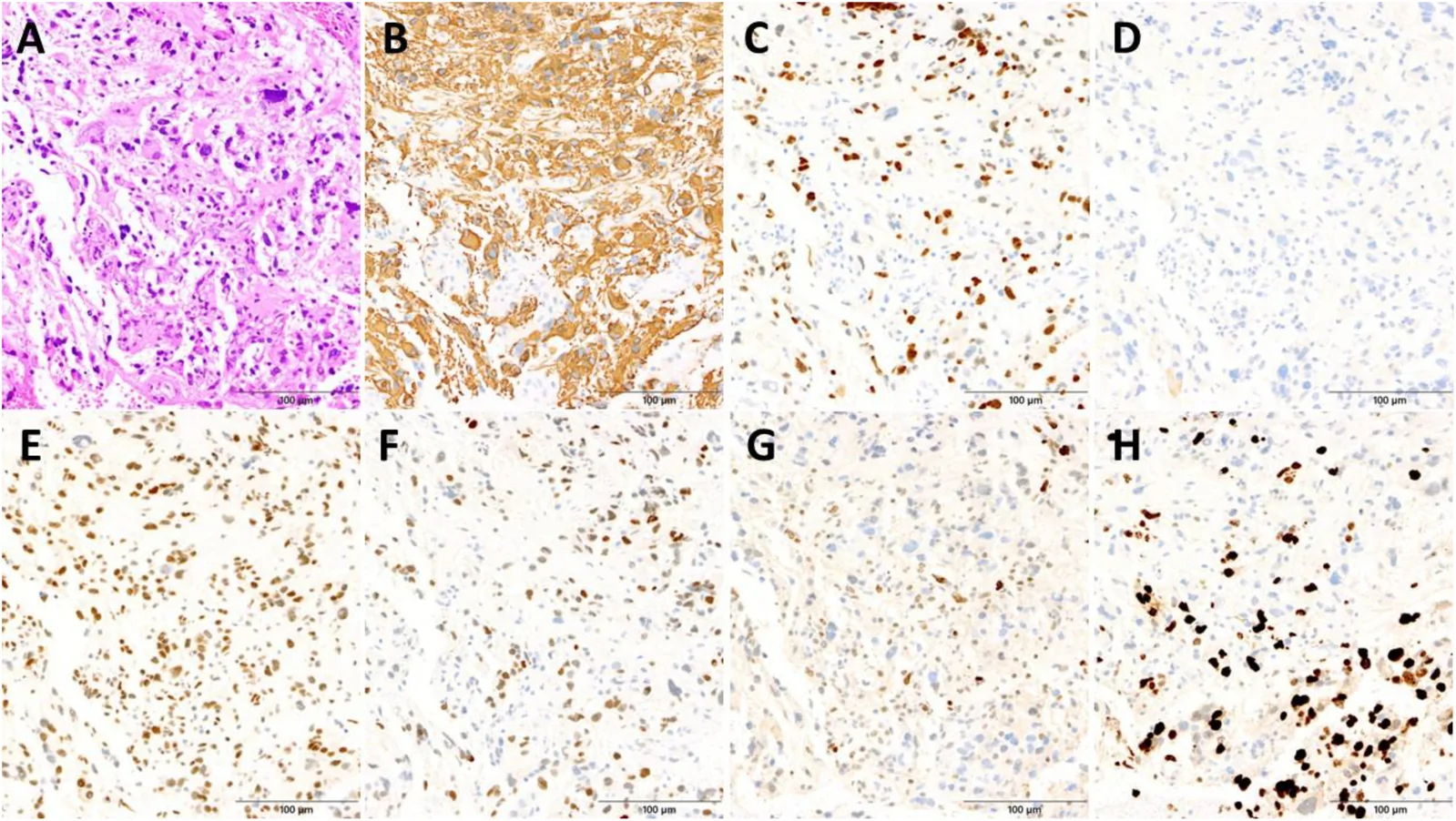
Histopathological findings. Hematoxylin-eosin staining demonstrates densely proliferating atypical astrocytic cells with microvascular proliferation and marked nuclear pleomorphism without tumor necrosis (A). Immunohistochemical staining shows positivity for GFAP (B) and Olig2 (C) and negativity for IDH1 R132H (D). Nuclear expression of ATRX is retained (E), and p53 shows overexpression (F). H3K27me3 expression is lost (G), and the Ki-67 labeling index is high (H). Abbreviations: ATRX, alpha-thalassemia/mental retardation syndrome X-linked; GFAP, glial fibrillary acidic protein; Olig2, oligodendrocyte transcription factor 2; IDH1, isocitrate dehydrogenase 1.

Histopathologic examination of the biopsy specimen revealed densely proliferating atypical astrocytic cells with microvascular proliferation, without evidence of tumor necrosis. Tumor cells demonstrated enlarged nuclei with increased chromatin, marked pleomorphism, including multinucleated cells and bizarre nuclei. Immunohistochemical analysis showed positivity for glial fibrillary acidic protein (GFAP) and oligodendrocyte transcription factor 2 (Olig2) and negativity for isocitrate dehydrogenase 1 (IDH1) R132H. Nuclear expression of alpha thalassemia/mental retardation syndrome X-linked (ATRX) was retained, and p53 showed overexpression. H3K27me3 expression was lost, and the Ki-67 labeling index was 51.5% (Fig. 2). Genetic analysis identified an H3F3A p.K28M (H3.3 K27M) mutation, whereas no canonical point mutations were detected in IDH1 or IDH2. Based on these findings, the tumor was diagnosed as diffuse midline glioma, H3 K27-altered (H3.3 K27M-mutant subtype), CNS WHO grade 4.

At autopsy, the tumor presented as a solid, whitish mass measuring 34 × 28 × 24 mm with focal necrosis, centered in the hypothalamus and the periventricular region of the third ventricle. This central mass almost completely replaced and destroyed the hypothalamus, bilateral medial thalami and medial optic tracts. Notably, while the mass itself was slightly eccentric to the left, the resulting tissue destruction was generally symmetric. The tumor filled the third ventricle and extended into the adjacent subarachnoid space, encasing the proximal segments of the bilateral internal carotid, middle cerebral, and anterior cerebral arteries.

Extensive progression from the central mass was evident in multiple directions. Rostrally, the tumor involved the bilateral orbital and straight gyri, as well as the inferior longitudinal fissure, directly infiltrating the optic chiasm and bilateral optic nerves.

Ventrally, the tumor protruded toward the pituitary region, infiltrating and destroying the posterior pituitary lobe. Dorsally it extended into the lateral ventricles, reaching the vicinity of the choroid plexus.

**Fig. 3:**
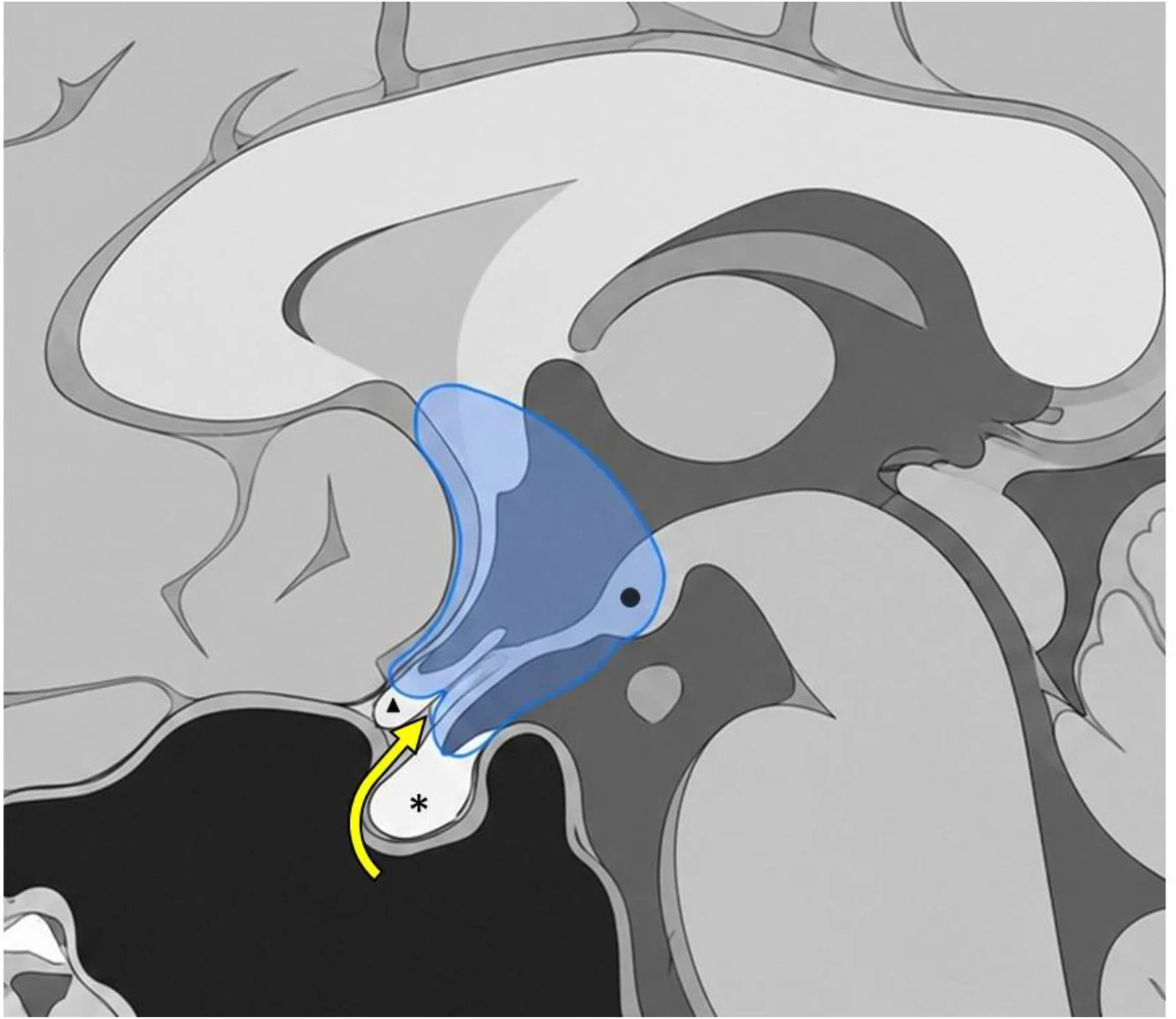
Schematic presentation of the autopsy findings. The tumor was centered in the hypothalamus and the periventricular region of the third ventricle, almost completely replacing these structures. The blue-shaded area indicates the extent of the tumor. The tumor was continuous with the optic chiasm (▲) rostrally, the mammillary body (●) caudally, the pituitary gland (*) ventrally, and the thalami and hypothalamus laterally. Notably, there was no direct continuity between the tumor at the inferior surface of the optic chiasm and the tumor infiltrating ventrally into the pituitary region (yellow arrow). The background brain anatomy was schematically illustrated with the assistance of ChatGPT, using a brain MRI provided by the first author as a reference.

Subpial aggregates of tumor cells were observed along the optic chiasm and the inferior surface of the optic tracts. This region was considered the invasive front, strongly suggesting that the tumor infiltrated the optic chiasm in a superior-to-inferior direction. Assessment of spatial continuity showed continuous tumor extension from the hypothalamus into the subarachnoid space; however, there was no direct continuity between the tumor at the inferior surface of the optic chiasm and the tumor mass infiltrating ventrally into the pituitary region (Fig. 3).

Although dissemination was not clearly evident on gross examination, microscopic evaluation revealed widespread involvement. Widespread dissemination was identified throughout the ventricular system and the subarachnoid space. Furthermore, the tumor exhibited extensive single-cell infiltration into the surrounding brain parenchyma, extending into the brainstem.

Gross examination of the lungs showed no significant findings, whereas microscopic evaluation revealed focal, minimal pneumonia in the left lower lobe.

## Discussion

Diffuse midline glioma, H3 K27-altered, is a diffuse glioma arising in midline locations and is classified as a pediatric-type diffuse high-grade glioma, CNS WHO grade 4, in the 2021 WHO Classification of Tumors of the Central Nervous System [1]. Loss of trimethylation of histone H3 lysine 27 caused by H3 K27M mutation and related mechanisms leads to dysregulation of transcriptional repression and contributes to tumorigenesis [1]. Regardless of histologic grade, these molecular alterations are associated with aggressive behavior and poor prognosis [2]. The entity was previously termed diffuse midline glioma, H3 K27M-mutant in the 2016 WHO classification; however, the nomenclature was revised after it was recognized that loss of H3 K27 trimethylation can occur through mechanisms other than H3 K27M mutation, including EZHIP overexpression [1].

Although DMG most commonly occurs in children and young adults, it has been reported across a wide age range, with a median age at diagnosis of 27 years (range, 4-76 years) [3]. The most frequent locations are the brainstem, spinal cord, and thalamus; brainstem involvement is more common in pediatric patients, whereas thalamic involvement predominates in adults [2,3].

Imaging findings of DMG are variable. Lesions may have well-defined or ill-defined margins; relatively well-defined margins are common, whereas extension beyond midline structures often results in irregular and indistinct borders [4]. Contrast enhancement ranges from absent to marked with central necrosis, and peritumoral edema is reportedly less prominent than in wild-type gliomas [4]. Pontine lesions may demonstrate the basilar artery wrapped sign [4]. Cystic degeneration and intratumoral hemorrhage are common, and fulminant hemorrhagic presentations have been reported [4]. Compared with wild-type gliomas, DMGs may show lower ADC values and higher rCBV [5]. Because of this imaging diversity, tumor location is an important clue to diagnosis.

In the present case, the initial manifestations were visual symptoms, without clear hypothalamic dysfunction, and MRI demonstrated a mass centered on the optic chiasm. Therefore, the lesion was initially considered to arise from the optic chiasm. The preoperative differential diagnoses for a mass involving the optic chiasm and adjacent structures included optic pathway gliomas (high-grade gliomas such as glioblastoma and DMG, as well as pilocytic astrocytoma), craniopharyngioma, and pituitary neuroendocrine tumor. Pilocytic astrocytoma and craniopharyngioma are typically benign tumors and exhibit well-defined margins. Additionally, pilocytic astrocytoma is common in pediatric patients with neurofibromatosis type 1 (NF1). Given the infiltrative growth pattern and rapid clinical course of the present case, these entities were considered unlikely. The patient's age and lack of a history of NF1 made pilocytic astrocytoma even less likely. A pituitary neuroendocrine tumor was also considered unlikely because the pituitary gland appeared normal on brain MRI. Although a pituitary neuroendocrine tumor arising from an ectopic pituitary gland remained in the differential diagnosis, its extreme rarity and the tumor’s extension along the optic tract made this diagnosis unlikely. Therefore, a high-grade glioma was considered the most likely diagnosis. Metastatic disease was also considered; however, no primary lesion was identified on whole-body CT.

The distribution of suprasellar tumors differs between age groups. In children, craniopharyngioma (50%), hypothalamic–chiasmatic gliomas, hypothalamic hamartomas, and germinomas are commonly reported [6]. In adults, pituitary adenomas (48%), suprasellar meningiomas (28%), and craniopharyngiomas (25%) are reported as the most frequent lesions [7]. DMG presenting as a sellar or suprasellar mass, as in the present case, is extremely rare, and only a limited number of such cases have been reported to date [8,9]. These reports are summarized in Table 1.

**Table 1:** Summary of case reports of diffuse midline glioma in the suprasellar region.

References	Age /sex	Primary location	Clinical presentation	MRI T1/T2 findings	Enhancement pattern	Other MRI features	Preoperative diagnosis	Surgical resection	Adjuvant therapy	Outcome
Present case	56, M	Optic chiasm /hypothalamus	Progressive visual impairment	T1: Heterogeneous hypointensity T2/FLAIR: Heterogeneous hyperintensity, partial cystic degeneration	Heterogeneous enhancement with central nonenhancing areas	Decreased ADC (0.98 × 10^−3^ mm^2^/s) Elevated rCBV	Suspected high-grade glioma	Subtotal resection (biopsy)	TMZ + IMRT (52 Gy)	Died at 5 mo
Wang et al. [9]	54, F	Third ventricle (suprasellar region)	Memory loss, gait instability	T1: Isointense T2: Mild T2 hyperintensity, cystic and solid components	Irregular annular enhancement, local lace-like enhancement	Bilateral ventricular dilatation Long T2 signal in paraventricular white matter	Ependymoma	Subtotal resection	TMZ + RT (60 Gy)	Alive at 11 mo
He et al. [8]	27, F	Hypothalamus (extending to suprasellar cistern)	Amenorrhea, polydipsia, diuresis, headache, lethargy	T1: Iso- to hypointense T2: Predominantly hyperintense	Strongly heterogeneous enhanced solid lesion and nonenhancing cystic lesion	Decreased ADC (1.28 × 10^−3^ mm^2^/s) and Increased rCBV (at recurrence)	Pituitary adenoma	Total resection (transsphenoidal) +Second surgery for recurrence	TMZ + RT (50 Gy)	Died at 9 mo

ADC, apparent diffusion coefficient; F, female; FLAIR, fluid-attenuated inversion recovery; IMRT, intensity-modulated radiation therapy; M, male; MRI, magnetic resonance imaging; rCBV, relative cerebral blood volume; RT, radiation therapy; T1, T1-weighted; T2, T2-weighted; TMZ, temozolomide.

To the best of our knowledge, no previous reports have described DMG originating from the optic tract. At autopsy, the main lesion was located in the hypothalamus and the periventricular region of the third ventricle, suggesting extension into the optic chiasm from above. Tumor continuity was present from the hypothalamus into the subarachnoid space; however, there was no direct anatomical continuity between the tumor beneath the optic chiasm and the tumor invading the pituitary region. Although the site of origin could not be established definitively, these findings favored a hypothalamic origin with exophytic growth forming a suprasellar mass and secondary invasion of the optic chiasm rather than a primary optic chiasm tumor.

Hypothalamic dysfunction can produce varied clinical manifestations, including changes in body mass index (BMI), symptoms of pituitary insufficiency, and disturbances of circadian rhythm, thermoregulation, and behavioral control [10]. Hypothalamic symptoms are reportedly absent in approximately 18.7% of patients diagnosed with suprasellar tumors [10]. In the present case, the fever shortly before death could have reflected hypothalamic thermoregulatory dysfunction but could also have been related to the concurrent minimal pneumonia. Thus, the clinical evidence of hypothalamic dysfunction was inconclusive. Moreover, lesions centered in the hypothalamus may initially present with visual impairment as the chief complaint [11]. Therefore, in the present case, despite the absence of prominent clinical signs of hypothalamic dysfunction and the initial presentation with visual field disturbance, a hypothalamic origin should be considered. Caution is warranted when inferring the primary tumor site solely from clinical presentation.

This case is notable for a DMG with an exophytic component involving the optic chiasm, an extremely rare presentation. DMG can be difficult to diagnose because of its diverse imaging appearances and may even mimic a low-grade glioma. Including DMG in the differential diagnosis of suprasellar masses may reduce the risk of overlooking this entity, facilitate prompt pathologic diagnosis, and allow the earlier initiation of treatment.

## Conclusion

We report a rare case of diffuse midline glioma, H3 K27-altered, presenting as a suprasellar mass with optic tract involvement. Although suprasellar location is uncommon, including DMG in the differential diagnosis of suprasellar masses may lead to early diagnosis.

## Declaration of generative AI and AI-assisted technologies in the manuscript preparation process

During the preparation of this work, the authors used Gemini and ChatGPT in order to proofread and improve the English text. ChatGPT was also used to assist in generating the background anatomical illustration for Figure 3, based on a brain MRI provided by the first author. The tumor extent and annotations were added and verified by the authors. After using these tools, the authors reviewed and edited the content as needed and take full responsibility for the content of the published article.

## Patient consent

Written informed consent was obtained from the patient's next of kin for the publication of this case report and any accompanying images.
